# Resection of posterior fossa tumors in the semi-sitting position in children younger than 4 years of age

**DOI:** 10.1007/s00381-022-05725-y

**Published:** 2022-11-09

**Authors:** Elvis J. Hermann, Gökce Hatipoglu Majernik, Dirk Scheinichen, Shadi Al-Afif, Hans E. Heissler, Thomas Palmaers, Joachim K. Krauss

**Affiliations:** 1 Department of Neurosurgery, Medical School Hannover, Carl-Neuberg-Str.1, 30625 Hannover, Germany; 2 Department of Anaesthesiology and Intensive Care, Medical School Hannover, Hannover, Germany

**Keywords:** Pediatric neurosurgery, Posterior fossa tumors, Semi-sitting position, Surgical technique, Venous air embolism

## Abstract

**Purpose:**

The semi-sitting position for resection of posterior fossa tumors is a matter of ongoing debate. Here we report about our experience with this approach in children younger than 4 years of age.

**Methods:**

We retrospectively analyzed data of children younger than 4 years of age operated on in our institution in the semi-sitting position over a 15-year period. Patients were intraoperatively monitored for venous air embolism (VAE) by transthoracic Doppler (TTD) or transesophageal echocardiography (TEE). The severity of VAE was classified according to the Tübingen grading scale. Intraoperative incidents of VAE were recorded and the patients’ course was followed postoperatively with a special focus on possible complications.

**Results:**

Twenty-four children (18 boys, 6 girls) were operated on in the semi-sitting position (26 operations). Mean age was 2.2 years (± 1.0), range between 0.4 and 3.9 years. External ventricular drains were inserted in 18 children with hydrocephalus preoperatively. VAE was detected in 6 instances during surgery (6/26 (23.1%)). In 3 patients with grade 1 VAE, no additional treatment was necessary. In one patient with grade 2 VAE, intracardiac air suction via the central venous catheter was performed, and in two patients with grade 4 VAE, additional cathecholamine-infusion was administered. No major intraoperative complications occurred. Postoperative CT images showed pneumocephalus in all children. In two children, small asymptomatic impression skull fractures at the site of the Mayfield pin occurred. Revision surgery was necessary in one child with a suboccipital CSF fistula.

**Conclusion:**

The semi-sitting position for resection of tumors in the posterior fossa in children younger than 4 years of age can be safely performed in experienced centers taking special caution to detect and treat potential complications in an interdisciplinary setting.

## Introduction

Positioning the patient for cranial tumor surgery is one of the most important steps with a high impact for a successful operation. The semi-sitting position for the resection of posterior fossa tumors has a long history and is an alternative to the prone or the lateral position [1–4]. It offers several surgical advantages such as easier anatomical orientation, gravity-aided drainage of irrigation and clearer vision, less necessity for suction, avoidance of venous congestion by cerebellar retraction, and reduction of the need for bipolar coagulation [1, 5–7]. It also provides easier access to the aqueduct and to the pineal region especially in tumors located in the midline of the posterior fossa [5, 8].

Furthermore, the cerebellomedullary fissure approach [9] to reach tumors of the fourth ventricle obviating the need for vermian splitting is easier to perform in the semi-sitting position. In the cerebellopontine angle, the identification and preparation of cranial nerves from tumor tissue can be achieved more smoothly allowing bimanual dissection [10, 11]. Compared to the prone position, the semi-sitting position has also advantages from an anesthesiological point of view allowing better and direct access to the airways, the bloodlines, and no compression of the chest like in the prone position [12].

Nevertheless, the semi-sitting position harbors some risks, with the most dreaded being venous air embolism (VAE) and arterial hypotension [6, 13]. VAE may occur in up to 38.6% [14] of surgeries depending on the measures used for its detection and its definition [1, 5, 6, 12, 13, 15, 16]. While the semi-sitting position has been abandoned in many centers because of its potential complications, there has been a renaissance, especially in European countries more recently [1, 2, 11, 12, 17–19].

There is limited experience with the semi-sitting position in children [1, 2, 4, 8, 19–22] especially in children younger than 4 years of age [1, 8, 19, 23]. Such surgery is a challenge even for an experienced team with regard to the thinness of the skull for rigid fixation [24], the small bodies to be positioned [23], and the particular anesthesiological circumstances [19, 22, 25]. Here we report about our experience with the semi-sitting position in a consecutive series of 24 children who were younger than 4 years at surgery operated within a period of 15 years.

## Methods

### Study population

A total of 236 children who were operated on for a brain tumor in our institution over a 15-year period were screened for surgeries of posterior fossa tumors. Inclusion criteria for the present study were (1) surgery in the semi-sitting position and (2) age younger than 4 years at the time of surgery. Demographic and clinical data were analyzed retrospectively according to patients’ charts and operative protocols. Intraoperative incidents were recorded and the patients’ postoperative course was evaluated with a special focus on possible complications due to the semi-sitting position. The parents gave consent for surgery and for using patients’ data for research purposes.

### Selection criteria for surgery in the semi-sitting position

At the time of presentation, preoperative MR images in all children with posterior fossa tumors had been analyzed by the first and the senior author to determine the best surgical approach and the positioning for surgery. If the semi-sitting position appeared to be the preferable option for surgery, a persistent foramen ovale was excluded by transthoracic echocardiography (TTE). A persistent foramen ovale with a hemodynamic relevant right to left shunt harboring the risk for paradox embolic complications and a very thin skull harboring the risk for an impressed skull fracture due to Mayfield clamp fixation were considered contraindications for the semi-sitting position, and these infants were operated in the prone position with fixation of the head on a horse-shoe head holder. The selection criteria for the semi-sitting position included (1) large tumors involving midline structures like the fourth ventricle, its surroundings, and the pineal region, and (2) tumors of the cerebellopontine angle (CPA).

### Anesthesiology protocol

General anesthesia was induced either with thiopentone 5–7 mg/kg or propofol 3–5 mg/kg lean body weight (LBW), sufentanil 0.5 μg/kg LBW, and atracurium 0.5 mg/kg LBW. After tracheal intubation, maintenance of anesthesia was achieved with propofol 8–10 mg/kg/h LBW and sufentanil 0.5–1.0 μg/kg/h LBW. All children were mechanically ventilated with oxygen/air. The ventilator minute volume was set to keep the partial pressure of arterial carbon dioxide (PaCO_2_) between 35 and 40 mmHg.

Positive end-expiratory pressure (PEEP) was kept at 4–5 cm H_2_O. Standard monitoring included electrocardiography (ECG), peripheral oxygen saturation (SpO2), noninvasive arterial pressure, etCO_2_, and body temperature. All patients received a central venous catheter (ArrowTM Blueguard 5.5 Fr, 3 lm, 6–13 cm, Teleflex, Reading, PA, USA) via the internal jugular or subclavian vein. The catheter tip was placed under ECG control (Arrow-JohansTM, Teleflex, Reading, PA, USA) in the right atrium. This was mandatory to have the possibility for suction of detected air bubbles if VAE occurred during surgery. The central line was placed always with real-time ultrasound control. A 24- or 22-G arterial line (ArrowTM-SAC-00524 or SAC-00522, Teleflex, Reading, PA, USA) was placed in a radial artery to measure arterial pressure. All patients received 10 ml/kg isotonic crystalloid infusion (Sterofundin ISO or Ringer-Lösung, BBraun, Melsungen, Germany) and 10 ml/kg colloid infusion (Gelafundin® 4%, BBraun, Melsungen, Germany) before being positioned in the semi-sitting position to maintain hemodynamic stability. Mean arterial pressure (MAP) was referenced to the tragus and kept between 50 and 60 mmHg according to age. To maintain an adequate MAP, crystalloids (5–10 ml/kg/h) were infused continuously, and when necessary, norepinephrine (0.01–0.05 μg/kg/min) was given.

To monitor VAE, either transthoracic Doppler (TTD, Model 915-BL, Parks Medical Electronics Inc., Aloha, OR, USA) or transesophageal echocardiography (TEE, Esaote MyLab25 Gold®, Köln, Germany) was used. TTD was placed at the fourth intercostal space on the left side just lateral to the sternum. Correct location was verified by injection of 5 ml saline via the central venous catheter and the occurrence of air bubbles. The TEE probe was positioned mid-esophageal to achieve a bicaval view.

### Treatment of concomitant hydrocephalus

In the presence of hydrocephalus, the need for an external ventricular drainage (EVD) was evaluated, and if considered necessary, it was inserted via a right frontal burr hole.

### Electrophysiology in the supine and the semi-sitting position

Intraoperative monitoring included medianus and tibialis SSEPs and AEPs. A baseline run for medianus SSEPs was recorded in the supine position and then once again in the semi-sitting position. If any impairment was detected, anteflexion of the head was reduced.

### Patient positioning

**Fig. 1:**
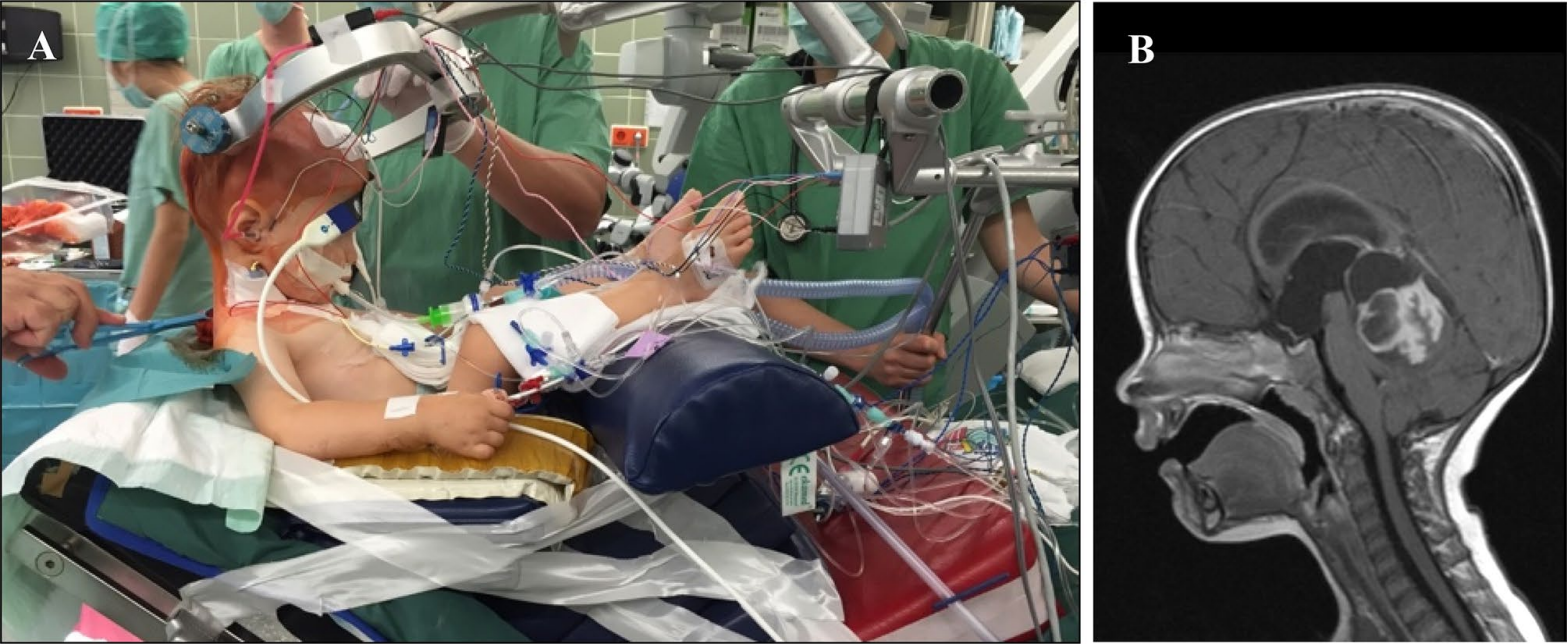
A 1.5-year-old infant in the semi-sitting position with the legs elevated up to the level of the transverse sinus to minimize the risk for VAE (**A**) for surgery of a pilocytic astrocytoma extending up to the pineal region (**B**)

The head was fixed in a three point fixation skull clamp (Mayfield Clamp, Integra Life Sciences) taking utmost care to avoid injury to the thin skull. The semi-sitting position was established thereafter according to a standard protocol (see more details published elsewhere, [1, 7, 8, 17, 26] with a neuroanaesthesiological team specialized in the semi-sitting position in pediatric patients. While the neurosurgeon held the head of the child fixed in the Mayfield clamp, the anesthesiologist monitored blood pressure and ventilation, and the OR nurse slowly elevated the upper part of the operating table. Once positioning in the semi-sitting position was achieved, the head was slightly flexed for midline tumors or rotated and flexed for CPA tumors. Additionally, surgical pads were placed as a “sitting bank” for perfect positioning, in particular in small infants. Anteflexion of the head permitted to place two fingers between the chin and the sternum to avoid venous congestion and to allow jugular vein compression during surgery. Figure 1 shows an example of a 1.5-year-old infant in the semi-sitting position for surgery of a tumor via a supracerebellar approach. The legs are positioned over the level of the transverse sinus to avoid negative venous pressure.

### Surgical approaches

All tumors involving the IVth ventricle were operated on via a telovelar approach, tumors of the CPA by a retrosigmoid approach, and tumors in the pineal region by an infratentorial/supracerebellar approach according to departmental standard techniques as outlined elsewhere [1, 8, 17, 26].

### Detection and management of intraoperative venous air embolism

VAEs were monitored either by TTD or TEE as outlined above. VAEs were classified for the present study according to the Tübingen VAE grading scale [15] (see Table 1).

**Table 1:** Classification of VAE according to the Tübingen VAE grading scale (according to Feigl et al., 2014)

Grade	TTD	TEE
0	No sounds, no VAE	No air bubbles, no VAE
1	Typical sound	Air bubbles visible
2	Typical sound and	Air bubbles visible and
	etCO_2_ decrease ≤ 3 mmHg	etCO_2_ decrease ≤ 3 mmHg
3	Typical sound and	Air bubbles visible and
	etCO_2_ decrease > 3 mmHg	etCO_2_ decrease > 3 mmHg
4	Typical sound and	Air bubbles visible and
	etCO_2_ decrease > 3 mmHg and	etCO_2_ decrease > 3 mmHg and
	MAP decrease ≥ 20% or	MAP decrease ≥ 20% or
	HR increase ≥ 40% or both	HR increase ≥ 40% or both
5	Typical sound and following CPR	Air bubbles visible and following CPR

*TTD* Transthoracic Doppler, *TEE* Transesophageal Echocardiography, *VAE* Venous Air Embolism; *etCO*_*2*_ End-tidal Carbon Dioxide, *MAP* Mean Arterial Pressure, *HR* Heart Rate, *CPR* Cardiopulmonary Resuscitation

### Statistics

Metric and nominal data were reported as mean ± standard deviation and percentage, respectively. Fisher’s exact test and binary logistic regression were used to clarify the dependencies on the incidence of VEA by possible risk factors. Fisher’s exact test was used to determine if there was a significant association between the occurrence of VAE concerning sex, age, weight, height, tumor type, tumor localization, tumor volume, the presence of preoperative hydrocephalus, the presence of preoperative EVD, the surgical approach, and the duration of surgery. Statistics were calculated using JMP®, version 16.2 (SAS Institute Inc., Cary, NC, 1989–2021).

## Results

According to the inclusion and exclusion criteria, a total of 24 children who underwent tumor resection in the semi-sitting position were identified (Table 2). This means that 1–2 cases were operated per year which constitutes about 2% of all posterior fossa tumors undergoing surgery annually. Two patients underwent surgery twice within an interval of 10 months (patient 21) and 11 months (patient 17) respectively, both for tumor recurrence. Thus, a total of 26 operations were performed. Note that 5 patients were also included in a previous study [1]. Overall, there were 18 boys and 6 girls with a mean age of 2.2 (± 1.0) years at the time of the first surgery. The youngest child was 0.4 years old and the oldest 3.9 years. Mean body weight was 13.2 (± 2.5) kilograms, ranging from 9 to 20 kg.

**Table 2:** Demographic data, imaging findings, and histopathological diagnosis in 24 children (< 4 years) with posterior fossa tumors operated in the semi-sitting position

Case no	Sex	Age at operation (years)	Bodyweight (kg)	Height (cm)	Tumor localization	Tumor size	Histopathology
1	m	3.6	15.6	101	IV. ventricle and CPA	4.0 × 2.0 × 1.8	Medulloblastoma
2	m	3.7	16.0	128	IV. ventricle	2.0 × 2.0 × 1.5	Medulloblastoma
3	m	3.1	14.0	110	IV. ventricle	5.0 × 3.5 × 3.4	Anaplastic ependymoma
4	m	1.4	11.0	80	Cerebellum/vermis	5.0 × 4.3 × 3.8	Glioblastoma
5	f	1.9	13.0	95	Cerebellum/vermis	5.8 × 5.2 × 4.8	Pilocytic astrocytoma
6	f	2.8	12.0	92	Middle cerebellar peduncle	2.0 × 1.3 × 1.2	Ganglioglioma
7	m	2.2	12.0	86	IV. ventricle	3.5 × 4.4 × 3.4	Medulloblastoma
8	f	1.7	11.0	85	Cerebellum/vermis	4.6 × 4.9 × 4.2	Medulloblastoma
9	f	2.0	11.0	83	Cerebellum/vermis	5.3 × 3.6 × 2.9	Pilocytic astrocytoma
10	f	1.2	14.0	90	IV. ventricle	4.9 × 4.1 × 3.8	Medulloblastoma
11	m	1.6	12.0	85	Cerebellar	5.9 × 4.2 × 4.0	Pilocytic astrocytoma
12	m	3.8	20.0	105	Cerebellum/vermis	4.2 × 4.0 × 3.7	Pilocytic astrocytoma
13	m	3.0	15.0	93	IV. ventricle	4.2 × 4.6 × 3.7	Medulloblastoma
14	m	2.8	15.0	98	Cerebellum/vermis	5.5 × 5.0 × 4.8	Pilocytic astrocytoma
15	m	0.4	9.0	63	IV. ventricle	3.8 × 1.9 × 1.6	Anaplastic ependymoma
16	m	3.9	17.0	107	CPA and foramina Luschkae	4.2 × 4.3 × 3.5	Pilocytic astrocytoma
17a	m	0.8	11.5	80	IV. ventricle and foramina Luschkae	1.1 × 0.7 × 0.5	Ganglioglioma
17b	m	1.7	15.0	94	Cerebellum/vermis	1.3 × 0.9 × 0.6	Ganglioglioma
18	f	3.1	13.0	97	CPA and brainstem	3.5 × 3.7 × 2.9	Anaplastic ependymoma
19	m	2.0	14.0	93	IV. ventricle	6.0 × 4.5 × 4.0	Medulloblastoma
20	m	2.2	13.7	92	IV. ventricle and foramina Luschkae	3.8 × 2.9 × 2.7	Anaplastic ependymoma
21a	f	2.0	10.0	71	IV. ventricle and middle cerebellar peduncle	2.9 × 3.7 × 2.5	Pilocytic astrocytoma
21b	m	1.7	10.0	81	Cerebellum/vermis	4.9 × 4.0 × 3.8	Pilocytic astrocytoma
22	m	3.7	15.0	96	IV. ventricle	5.4 × 5.6 × 5.0	Medulloblastoma
23	m	1.3	11.0	90	IV. ventricle and pinealis region	5.9 × 4.2 × 3.8	AT/RT
24	f	2.8	13.0	91	IV. ventricle and middle cerebellar peduncle	2.8 × 2.7 × 2.5	Pilocytic astrocytoma

*CPA* Cerebellopontine Angle, tumor size: largest diameter in 3 axes in cm, *AT/RT* Atypical Teratoid/Rhabdoid Tumor

The histopathological diagnoses included pilocytic astrocytoma (*n* = 8), medulloblastoma (*n* = 8), anaplastic ependymoma (*n* = 4), ganglioglioma (*n* = 2), glioblastoma (*n* = 1), and atypical teratoid rhabdoid tumor (*n* = 1). Tumor sizes for the individual patients are shown in Table 2. The mean tumor size was 30.2 (± 22.3) cm^3^.

External ventricular drains were inserted in 18 (69.2%) of the 26 surgeries directly preoperatively. Table 3 shows individual patient data, the surgical approaches used intraoperative findings, and the duration of surgeries. The mean time of surgery was 5.1 ± 1.4 h (2.6–8.3 h).

**Table 3:** Surgical data and postoperative findings in 24 children (< 4 years) with posterior fossa tumors operated in the semi-sitting position

Case No	Sex	Age at surgery (years)	Preoperative hydrocephalus	EVD preop	Duration of surgery (h:min.)	Surgical approach	Monitoring for VAE	Occurrence of VAE (Tübingen grade)	Intraoperative therapy for detected VAE	Postoperative pneumocephalus
1	m	3.6	Yes	Yes	3:52	Lateral suboccipital	TTD	None	-	Ventricles + + , SD +
2	m	3.7	No	No	3:55	Medial suboccipital	TTD	None	-	Ventricles + + , SD +
3	m	3.1	Yes	Yes	5:24	Medial suboccipital	TTD	None	-	Ventricles + +
4	m	1.4	Yes	Yes	4:28	Medial suboccipital	TTD	None	-	Ventricles + , SD + +
5	f	1.9	Yes	Yes	3:20	Medial suboccipital	TTD	Grade 4	Catecholamine-infusion, intracardiac suction of air	SD + +
6	f	2.8	No	No	2:38	Medial suboccipital	TEE	Grade 1	-	SD +
7	m	2.2	Yes	Yes	7:43	Medial suboccipital	TTD	None	-	Ventricles + +
8	f	1.7	Yes	No	2:33	Medial suboccipital	TTD	None	-	SD +
9	f	2.0	Yes	Yes	5:05	Medial suboccipital	TTD	None	-	SD + +
10	f	1.2	Yes	Yes	6:43	Medial suboccipital	TTD	None	-	Ventricles + +
11	m	1.6	Yes	No	5:20	Medial suboccipital	TTD	Grade 4	Catecholamine-infusion, intracardiac suction of air	SD + +
12	m	3.8	Yes	Yes	5:30	medial suboccipital	TTD	None	-	SD + +
13	m	3.0	Yes	Yes	5:21	Medial suboccipital	TTD	None	-	Ventricles + +
14	m	2.8	Yes	Yes	6:14	Medial suboccipital	TTD	None	-	SD + +
15	m	0.4	Yes	Yes	4:56	Medial suboccipital	TTD	None	-	Ventricles +
16	m	3.9	No	No	5:58	Lateral suboccipital	TEE	None	-	Ventricles + , SD +
17a	m	0.8	No	No	8:20	Medial suboccipital	TTD	Grade 1	-	SD + +
17b	m	1.7	No	No	6:35	Medial suboccipital	TTD	None	-	SD + +
18	f	3.1	Yes	No	4:18	Lateral suboccipital	TTD	None	-	SD + +
19	m	2.0	Yes	Yes	5:09	Medial suboccipital	TTD	None	-	Ventricles + + , SD +
20	m	2.2	Yes	Yes	4:25	Medial suboccipital	TTD	None	-	Ventricles + + , SD +
21a	f	2.0	Yes	Yes	3:52	Medial suboccipital	TEE	Grade 1	-	SD +
21b	m	1.7	Yes	Yes	4:23	Medial suboccipital	TTD	None	-	Ventricles + , SD + +
22	m	3.7	Yes	Yes	6:00	Medial suboccipital	TEE	None	-	Ventricles + + , SD +
23	m	1.3	Yes	Yes	6:37	Medial suboccipital	TTD	Grade 2	Intracardiac suction of air	Ventricles + + , SD +
24	f	2.8	Yes	Yes	4:40	Medial suboccipital	TEE	None	-	Ventricles + +

*VAE* Venous Air Embolism, *TTD* Transthoracic Doppler, *TEE* Transesophageal echography, *EVD* External Ventricular Drainage, *SD* Subdural, + + marked, + moderate/mild

VAE was detected in 6 instances during surgery (6/26 (23.1%)) and was classified as grade 1 in 3 patients (11.5%) according to the Tübingen grading scale, grade 2 in one patient (3.9%), and grade 4 in two patients (7.7%). VAE was detected by TTD in 4 instances and by TEE in 2. In the three patients with grade 1, VAE did not result in any cardiovascular or respiratory signs or symptoms. In the patient with VAE grade 2, air bubbles were removed from the right atrium by suction via the central venous line. In the 2 patients with VAE grade 4, surgery was briefly interrupted, the head of the table was lowered, air was removed from the right atrium, and catecholamines were given for the remainder of the surgery. The neurosurgical and the neuroanesthesiological teams considered to abandon surgery, but then decided to continue upon stabilization of the situation. There was no postoperative morbidity related to the occurrence of VAE.

Postoperative CT images showed pneumocephalus either in the ventricles or in the subdural space in all instances (Table 3). Three patients (11.5%) had moderate subdural air depots; 8 patients (30.8%) marked subdural air depots. One patient had moderate ventricular air depots; 5 patients (19.2%) had marked ventricular air depots. Eight patients (34.6%) had a combination of intraventricular and subdural air depots. Patient 4 with a glioblastoma and combined air depots developed a subdural hygroma and was treated by a permanent subduroperitoneal shunt 5 weeks after tumor surgery.

Postoperative complications related to tumor surgery occurred in 8 instances and included facial palsy (4 instances), ataxia (12 instances), and cerebellar akinetic mutism (5 instances). All these symptoms improved during hospital stay. There was no mortality. In two children, minor asymptomatic skull impression fractures at the site of a Mayfield pin were evident in the postoperative scans without a need for surgical intervention. Revision surgery was necessary in one child with a suboccipital CSF fistula. Five children needed permanent CSF diversion because of persisting hydrocephalus and underwent ventriculoperitoneal shunting subsequently. There were no complications due to the placement of the central venous catheter.

No statistically significant associations (two-sided) were found between possible risk factors and the occurrence of VAE except for age and weight (Table 4).

**Table 4:** Venous air embolism and possible risk factors

Venous air embolism	*P*
Sex	.3301
Age	**.0099**
Weight	**.0390**
Height	.1438
Tumor type	.0652
Tumor localization	1.00
Tumor volume	.9480
Presence of preoperative hydrocephalus	.5581
Presence of preoperative EVD	.3301
Surgical approach	1.00
Duration of surgery	.8720

Numbers in bold indicates significance

## Discussion

Our study shows that tumors of the posterior fossa can be operated safely with few and manageable intraoperative complications in the semi-sitting position in the very young age group when surgery is performed by experienced neurosurgical and anesthesiological teams. To our knowledge, this is the first study focusing on children younger than 4 years of age operated in the semi-sitting position.

Posterior fossa tumors are the most common brain tumors in childhood, representing about 2/3 of all pediatric brain tumors [27, 28]. Surgery is usually the first step in the treatment of such tumors and in benign tumors sometimes the only treatment modality. Therefore, maintenance and improvement of quality of life when performing surgery should be the highest priority. In small children and infants, like in our study, great care has to be taken during surgery considering the small circulating blood volume, the poor thermoregulation, and the incomplete maturation of brain, skull, and soft tissue [25]. The potential complications of surgery in the semi-sitting position have been sufficiently reported in adults and in older children [1, 6, 7, 11–15, 17–22, 29] but the age group of 4 years or younger has not been in the focus of attention.

Harrison et al. were among the first to report on using the semi-sitting position in a larger series of children. The incidence of VAE was 9.3% in a series of 407 children [22]. In a newer study including 97 children younger than 18 years operated in the semi-sitting position for posterior fossa tumors, VAE occurred in 21.6% and hemodynamic instability in 12.3%. The mean age in that study was 11.2 ± 4.5 years. VAE was noted in 20% in children < 9 years and in 22% in children > 9 years [21]. The overall rate of severe VAE including hypotension (5 patients) and desaturation (4 patients) was 9.3%. The overall safety of the semi-sitting position in children was also confirmed by a recent study including 38 children at a mean age of 8.9 years [19]. VAE grade 1 was detected in four cases and grade 2 in one case (11.9%) without clinical consequences. In 3 instances, severe venous sinus bleeding occurred during surgery (7.1%).

It appears that with exception of VAE and pneumocephalus complication rates are similar in pediatric neurosurgery for posterior fossa tumors when the semi-sitting position is compared to the prone position [2, 4, 19].

Orliaguet et al. compared 60 children operated on in the semi-sitting position (5.5–9 years old, weight 19–25 kg) versus 19 children operated in the prone position (2.2–11 years old, weight 15–30 kg) regarding complications. A serious VAE occurred in one patient in the semi-sitting position (2%) and was managed without postoperative complications. In the prone position, children received a larger median volume of intraoperative blood transfusion in contrast to children operated in the semi-sitting position [4].

There was also no difference in complication rates in a recent pediatric series operated for posterior fossa lesions when the semi-sitting position (42 surgeries) was compared with the prone position (24 surgeries) [19]. However, again there was a higher likelihood to have blood transfusions for those who were operated in the prone position.

Surgery in the semi-sitting position has a long tradition in our department [1, 8, 17, 26], especially for vestibular schwannoma surgery [1, 7, 17]. We have established algorithms to position the patient in a safe way which demands a concerted workflow of an experienced interdisciplinary team consisting of the neurosurgeon, the neuroanesthesiologist and the operative nurses. In a previous study, we have reported on 740 patients operated in the semi-sitting position [1]. In that study, there were no significant differences in the incidence of VAE in 687 adults (15.9%) versus 53 children (18.9%). TEE detected VAE in 40.5% of surgeries and TTD in 11.8%.

With that regard, it has to be noted that TEE has a higher sensitivity than TTD detecting more clinically irrelevant VAEs. In another study, TEE monitoring detected VAE in 37% versus TTD in 10% of surgeries [12]. Similar results were also reported by Ganslandt et al., with an occurrence of VAE of 26% when monitored with TEE and 9% with TTD [6].

TEE is practicable easily in children with a minimum of 10-kg bodyweight. The sensitivity of TTD appears to be higher in children because of the better acoustic windows related to their smaller size and thinner bodies. In our present study, in children younger than 4 years of age, both grade 4 VAEs were detected by TTD. It may be debated whether these VAEs would have been detected earlier with TEE and whether their severity could have been prevented.

There are few previous studies which compared the occurrence of VAE in the semi-sitting position in adults versus in children [1, 12, 20]. In a study with a total of 430 patients (334 adults and 96 children older than 5 years) operated in the semi-sitting position for posterior fossa surgery, the incidence of VAE and associated hypotension were analyzed [20]. There was a 28% incidence rate of VAE in adults, and a 22% rate in children without a significant difference.

According to the findings of Ibawuchi et al., smaller children might have a lower overall risk to develop VAE than adults [30]. Their study examined the pressure in the confluens sinuum under various conditions in 47 cases including 11 children. Especially in the sitting position, adults showed a negative venous pressure, whereas all 8 children less than 9 years of age had a positive pressure.

A relatively frequent complication of the semi-sitting position is pneumocephalus. As would be expected, pneumocephalus occurs significantly more often after surgeries in the semi-sitting positions (76.2%) than in the prone position (45.8%) [19]. Postoperative pneumocephalus was observed basically in all patients in our series. Since a ventricular tension pneumocephalus might require emergency EVD placement [18], we routinely implanted an EVD prior to tumor resection to manage the expected intraventricular air entrapment postoperatively.

In our series, there were two depressed but asymptomatic skull fractures caused by the sharp head fixation with the Mayfield clamp. Interestingly, a technical note by Muzumdar [23] described a custom-designed chair and its suitability for sitting position in infants to avoid sharp head fixation, however, not without limitations for the safety of the surgery.

A disadvantage of surgery in the semi-sitting position is the occurrence of brain shift which limits the use of neuronavigation [26]. Also, the infants need to be repositioned for intraoperative MR or CT imaging. Application of intraoperative real-time ultrasound is still possible and reliable; however, it requires greater experience.

## Conclusion

The semi-sitting position for resection of tumors in the posterior fossa in children younger than 4 years of age can be safely performed taking special caution to detect and treat potential complications early in an interdisciplinary setting. Despite its potential complications, it is a valuable alternative to the prone position.

## Data Availability

The datasets generate during and/or analyzed during the current study are available from the corresponding author on reasonable request.
